# Drug Screening for Discovery of Broad-spectrum Agents for Soil-transmitted Nematodes

**DOI:** 10.1038/s41598-019-48720-1

**Published:** 2019-08-26

**Authors:** Mostafa A. Elfawal, Sergey N. Savinov, Raffi V. Aroian

**Affiliations:** 1 Program in Molecular Medicine, University of Massachusetts Medical School Worcester, Worcester, USA; 2 Department of Biochemistry and Molecular Biology, University of Massachusetts Amherst, Amherst, USA

**Keywords:** Drug discovery, Drug screening, Phenotypic screening

## Abstract

Soil-transmitted nematodes (STNs), namely hookworms, whipworms, and ascarids, are extremely common parasites, infecting 1–2 billion of the poorest people worldwide. Two benzimidazoles, albendazole and mebendazole, are currently used in STN mass drug administration, with many instances of low/reduced activity reported. New drugs against STNs are urgently needed. We tested various models for STN drug screening with the aim of identifying the most effective tactics for the discovery of potent, safe and broad-spectrum agents. We screened a 1280-compound library of approved drugs to completion against late larval/adult stages and egg/larval stages of both the human hookworm parasite *Ancylostoma ceylanicum* and the free-living nematode *Caenorhabditis elegans*, which is often used as a surrogate for STNs in screens. The quality of positives was further evaluated based on cheminformatics/data mining analyses and activity against evolutionarily distant *Trichuris muris* whipworm adults. From these data, two pairs of positives, sulconazole/econazole and pararosaniline/cetylpyridinium, predicted to target nematode CYP-450 and HSP-90 respectively, were prioritized for *in vivo* evaluation against *A*. *ceylanicum* infections in hamsters. One of these positives, pararosaniline, showed a significant impact on hookworm fecundity *in vivo*. Taken together, our results suggest that anthelmintic screening with *A*. *ceylanicum* larval stages is superior to *C*. *elegans* based on both reduced false negative rate and superior overall quality of actives. Our results also highlight two potentially important targets for the discovery of broad-spectrum human STN drugs.

## Introduction

Soil transmitted nematodes (STNs) of humans- the giant roundworm *Ascaris lumbricoides*, whipworm *Trichuris trichiura*, and three hookworm species *Necator americanus*, *Ancylostoma duodenale*, and *Ancylostoma ceylanicum* –are intestinal parasites responsible for much of the morbidity associated with the neglected tropical diseases that sustain the cycle of poverty^1,2^. An estimated 438.9, 819, and 646.6 million people are infected with hookworms, giant roundworms, and whipworms, respectively, and, together, these parasites are responsible for at least 4.98 million years lived with disability YLDs^3^. Morbidity associated with STN infections include growth stunting, malnutrition, anemia, and cognitive impairment^1,4,5^. Of all these parasites, hookworms outrank the other STNs in terms of morbidity and are associated with significant contributions to anemia in children, pregnant women and adults^5,6^. *A*. *ceylanicum* is a zoonotic hookworm species that infects humans, cats, and dogs and a prevalent hookworm parasite in humans in Southeast Asia^7–11^.

To combat STNs, the World Health Organization (WHO) has recommended a long-term plan to improve sanitation and health education to limit infection, as well as routine Mass Drug Administration (MDA) campaigns targeting pre-school and school-aged children to eliminate morbidity^12^. Currently two benzimidazole drugs, namely, albendazole and mebendazole, originally developed for farm animal use, are adopted by the WHO in MDA against human parasites. Unfortunately, drug resistance against benzimidazoles and other anthelmintics is well established in veterinary parasites^13–17^. Benzimidazole resistance alleles have been found in human STNs and their increased frequency following anthelmintic treatment has been observed^18–20^. There are also multiple reports indicating low efficacy of albendazole against STNs^21–25^, and good single-drug efficacy against whipworms is lacking^26–28^. New screens for potent anthelmintic drugs with broad specificity and new mechanisms of action are thus urgently needed. Phenotypic screening, also called “whole organism screening,” remains an essential and productive approach for drug discovery, including for anthelmintics^29–31^. Target-based approaches, in contrast to phenotypic screening, require significant understanding of parasite biology (*e*.*g*., is the target essential? accessible to a given drug?), which is limiting for STNs. In phenotypic screening, identification of positives does not require the same amount of knowledge about the parasite but rather relies on the ability to test many molecules covering large area of chemical space.

Phenotypic drug screens against STNs have lagged behind that of many other diseases because of the difficulty in getting large quantities of relevant stages of STNs for screening and the relative dearth of studies testing and validating an appropriate screening system. Screens to date include using adult parasites *in vitro*^32,33^, eggs hatch assay^22,34^, larval-staged parasites^33–35^, infective third stage parasitic larvae (L3i)^36,37^, and the free-living nematode *Caenorhabditis elegans* as a surrogate^33,38–42^. Despite these studies, little groundwork has been done to determine the comparative validity and usefulness of such screens towards finding an anthelmintic against STNs that is efficacious *in vivo* in laboratory models or that would be useful in human therapy. In addition, there have been little studies done regarding optimization of even a moderate throughput pipeline for gastrointestinal nematode drug discovery while also focusing on or addressing the false positive and false negatives of the screening models used (*e*.*g*., the drug screens with adults above investigated at most 400 compounds).

To address these limitations while also performing a screen for effective anthelmintics, we screened to completion a 1,280-compound approved drug library (FDA, EMA and other agencies) against *A*. *ceylanicum* hookworm adults and egg/larval stages *in vitro* and against *C*. *elegans* fourth larvae (L4)/adult and egg/larval stages. Half of the FDA library (640 drugs) was also tested against regular and exsheathed *A*. *ceylanicum* L3i. Hookworm adult positives were further subjected to screening against whipworm adults, as well as cheminformatics and data mining analyses for down selection and prioritization. These analyses resulted in four candidate drugs that were taken forward for *in vivo* testing against hookworms in infected hamsters. Based on these data, we (i) propose a novel screening pipeline for STN drug discovery, (ii) identify an antiparasitic with heretofore unknown efficacy against hookworms based on parasite *in vivo* fecundity, and (iii) report on the potential identification of two putative targets for broad-spectrum STN therapy.

## Results

### Screening of an approved drug library against hookworm adults

Since adult parasites are the primary *in vivo* therapeutic target for anti-STN drugs and since large-scale drug screening cannot be reasonably carried out *in vivo* using infected rodents, we chose adult parasite *in vitro* screening as our standard against which all other screens would be compared. Since the human-parasitic hookworm *A*. *ceylanicum* can be maintained in immunocompetent hamsters, it is a relevant parasite to test as an actual human parasite in the laboratory.

**Figure 1:**
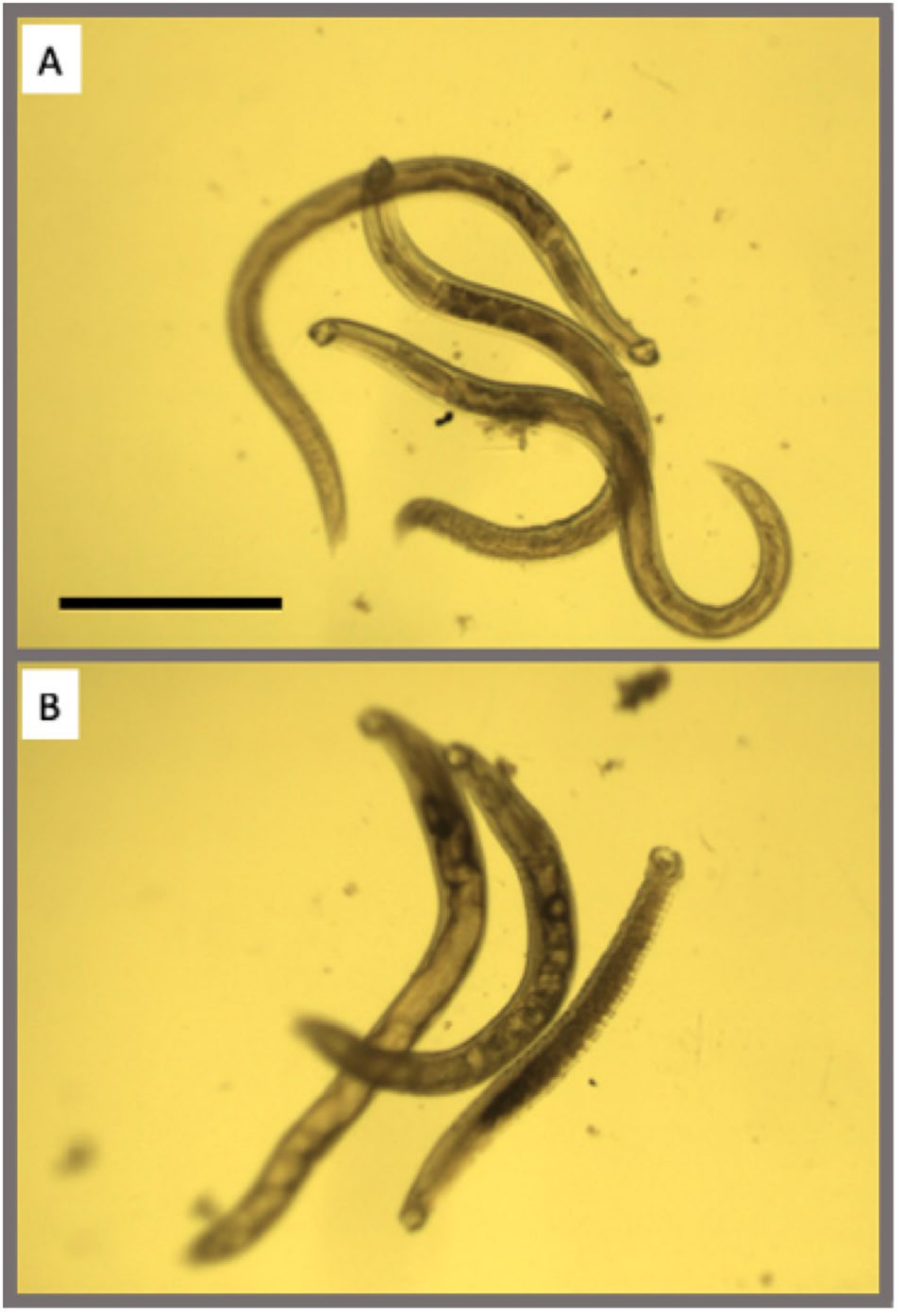
Example of screening with adult *A*. *ceylanicum* hookworms in 96 well plate. (**A**) Three adult hookworms in DMSO control well showing healthy parasites with normal gut morphology; (**B**) Three adult hookworms in well with active compound; animals are dead or shrunken, showing abnormal gut morphology. Scale bar here and elsewhere = 1 mm.

To establish which compounds in the library have activity against adult hookworms, all 1280 compounds were screened against *A*. *ceylanicum* adults and scored for impacts on motility and morphology (Fig. 1A,B). Out of the1280 drugs, 39 were found to be effective against adult *A*. *ceylanicum* parasites with ~3% hit rate (Table S1). Those 39 drugs span a diverse range of established biological activities, including analgesic, antibacterial, antifungal, antipsychotic, and antianginal. The screen was validated by selecting two of the 96-well plates (160 compounds)–one with the highest number of actives (6) and one with no actives in the initial screening, and rescreening them with adult *A*. *ceylanicum* parasites. There was perfect correspondence for compounds that were considered active and inactive in both screens.

### Screening of library using free-living *C. elegans* and free-living stages of *A. ceylanicum*

In order to test for convergence between various models with our adult hookworm standard, we next screened the library to completion using two *C*. *elegans* stages and the free-living egg-to-larval (E2L) stages of *A*. *ceylanicum* hookworms. By screening the entire library against these potential surrogates, we can determine both the false positive rate (actives that kill or intoxicate the surrogate but not the adult hookworm standard) and the false negative rate (actives that kill or intoxicate the adult hookworm standard but not the surrogate). The false negative rate has generally been poorly characterized in anthelmintic discovery studies but is important since it reveals if excellent actives might be missed by using an inadequate surrogate. We screened the entire library using different stages of *C*. *elegans* since different stages have been preferred in previous studies.

**Figure 2:**
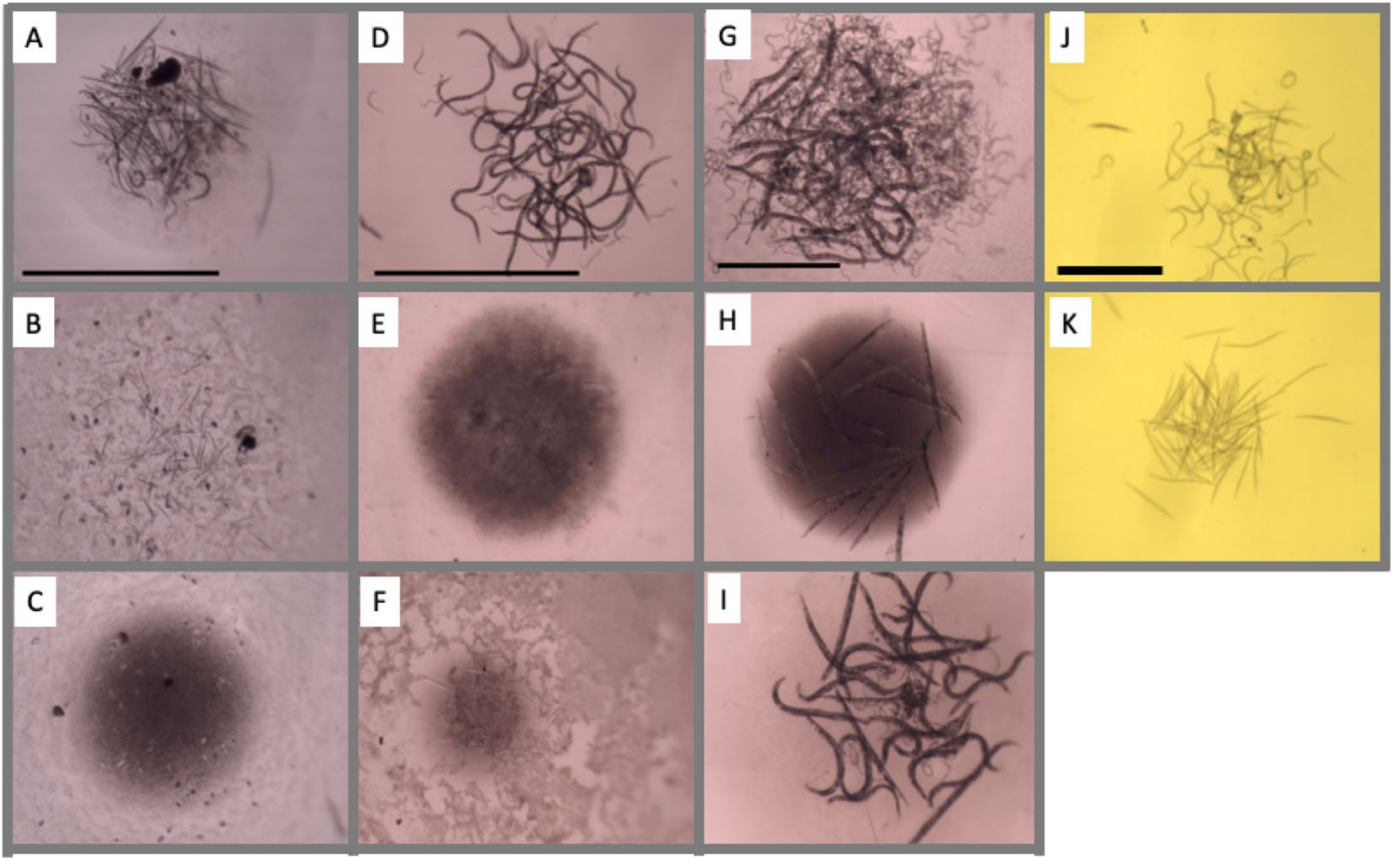
Phenotypic examples of actives in *A*. *ceylanicum* E2L screen (**A–C**), *C*. *elegans* E2A screen (**D–F**), *C*. *elegans* L4-Adult screen (**G–I)** and *A*. *ceylanicum* xL3i screen (**J,K**). (**A,D,G,J**) Showing DMSO control with healthy nematodes, including normal morphology (all), development (**A,D**), and fecundity (**G**). Motility was also normal in all wells. (**B,C,E,F,H,I,K)** Examples of actives with arrested or stunted growth (**B,C,E,F**), dead *C*. *elegans* L4/adults (**H**), sterile *C*. *elegans* (**I**), or dead/paralyzed xL3i’s (**K**).

For screening against the free-living stage of *A*. *ceylanicum* hookworms, eggs were isolated from hookworm-infected hamsters, cleaned up, and dispensed into 96 well-plates with an *Escherichia* *coli* food source. Over the course of 7 days, on average 85% of these eggs hatch and the resulting larvae grow and develop to the L3i stage in the absence of any drug (at which point they require a host to continue normal development). This 96-well plate assay, which we call egg-to-larva (E2L), recreates the soil/environmental stage of hookworms. Examples of screening with this E2L assay are shown in Fig. 2A–C. We set up a parallel egg-to-adult (E2A) assay with *C*. *elegans*, plating eggs in a 96-well format and allowing them to develop for 3 days until the adult stage was reached (Fig. 2D–F). As a parallel *C*. *elegans* assay for hookworm adults, we distributed late-staged *C*. *elegans* larvae (fourth stage or L4) in a 96-well format that, over the course of four days, develop into adults with significant brood sizes (Fig. 2G–I). The library was screened to its entirety with hookworm E2L, *C*. *elegans* E2A, and *C*. *elegans* L4s. The DMSO concentration was kept to levels that did not affect control (no drug) wells. Each was screened at 30 µM, as was done for adult hookworms. Since earlier stages tend to be more sensitive to drug than later stages, we also screened hookworm E2L and *C*. *elegans* E2A at 10 µM in order to compare results at multiple doses.

**Figure 3:**
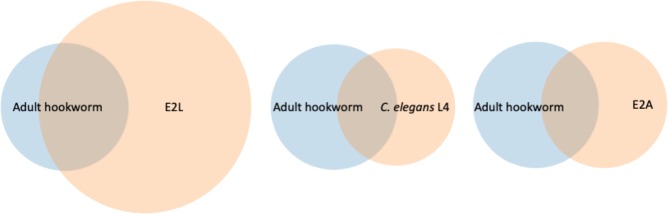
Actives Venn diagram. Shown schematically are the number of actives discovered using each of *A*. *ceylanicum* E2L, *C*. *elegans* L4 and *C*. *elegans* E2A overlapping with the 39 *A*. *ceylanicum* adult standard actives. Areas are representative of actual overlap rations. *A*. *ceylanicum* E2L showed the highest number of overlapping actives (27 of 39) with *A*. *ceylanicum* adults standard actives and the lowest percentage of the false negatives compared to *C*. *elegans* L4 and E2A.

The number of total actives and inactives found in all these screens and their overlap with the adult hookworm standard actives are indicated Table 1 and graphically in Fig. 3. The success rate (“true positive rate” or TPR) of each model was calculated as the number of actives overlapping with adult *A*. *ceylanicum* (Fig. 3). In order from the most successful to least successful we found: *A*. *ceylanicum* E2L (30 µM) 69% TPR, *A*. *ceylanicum* E2L (10 µM) 46% TPR, *C*. *elegans* L4 (30 µM) 36% TPR, *C*. *elegans* E2A (30 µM) 28% TPR, and *C*. *elegans* E2A (10 µM) 18% TPR. Which of the adult hookworm standard 39 positives overlapped with those from other screening methods is shown in Table S1.

**Table 1:** The total number of actives from each model tested, the number of overlapping hits with the hookworm (HW) *A*. *ceylanicum* adult standard actives, and the number of false positives and false negatives.

	HW adults 30 µM	HW E2L 30 µM	*C*. *elegans* L4 30 µM	*C*. *elegans* E2A 30 µM	HW L3i 30 µM	HW XL3i 30 µM	HW E2L 10 µM	*C*. *elegans* E2A10 µM
Total actives	39	108	34	29	7 of 640	9 of 640	58	15
Overlapping with HW adults	.	27	14	10	7 of 20	7 of 20	18	7
Non-HW adult (false positives)	.	81	20	19	0	2	41	8
Missed HW adult (false negatives)	.	12	25	28	13 of 20	13 of 20	21	32

### Screening the library with L3i and xL3i hookworm parasites

Infectious staged larvae (L3i) have also been proposed as a screening method^36,37,43^. Getting large quantities of these is a relatively straightforward task and, for some veterinary parasites, like *Haemonchus contortus*, extremely large quantities can be cultured from a single infected animal (*e*.*g*., sheep) for extended periods of time. However, this stage of parasitic nematodes are covered in a protective sheath and metabolically dormant, awaiting a host to exsheath and become active again^44,45^. As such, it has been proposed that exsheathed larvae (xL3) may be a better model for anti-nematode drug screening^33,36^. To test the utility of L3i or induced xL3i for drug screening, 640 compounds from the library were also screened against these stages. As indicated in Table 1, we found that these stages, like *C*. *elegans*, tended to give more false negatives than hookworm E2L, although the number of false positives was very low.

### Data mining and chemoinformatic characterization of hits

We also set out to determine the quality of the hits that each screening system produced. The quality of the hits were established in two ways. First, we used data mining and cheminformatics to characterize the 39 actives against adult hookworm parasites (Table S2). The 39 actives were split into two groups—those that were active against parasitic nematode, *A*. *ceylanicum*, but not the free-living nematode, *C*. *elegans* (Parasite-specific or PS) and those that were active against both the parasitic and free-living nematodes (Nematode-General or NG). The performance of PS and NG sets in the anthelmintic screen was compared to the performance of the same compounds in cytotoxicity assays deposited into the PubChem database. The two PubChem assays that screened the largest number of these compounds (33 and 30, respectively) are those against chicken DT40 (AID743012) and human HEK293 (AID1224886) cell lines. Of the compounds that were evaluated in these assays, 54% (7/13) and 40% (4/10) of NG actives had IC50 values at a submicromolar level in AID743012 and AID1224886, respectively, whereas the values for the PS hits were 15% (3/20) and 0% (0/19), respectively. At the same time, the percentages of moderately cytotoxic and non-cytotoxic compounds (IC50 ≥ 10 µM) were 23% and 36% for NG and 50% and 63% for PS. Although the sample size is admittedly limited, it is, nonetheless, notable that the smaller subset of PS-adult hits discovered via E2L screen also displays this reduced cytotoxicity trend. Thus, the IC50 values in the submicromolar range were shown by 2 (out of 10) and 0 (out of 10) such compounds in AID743012 and AID1224886 cytotoxic screens, respectively.

### *In vitro* whipworm screening

**Figure 4:**
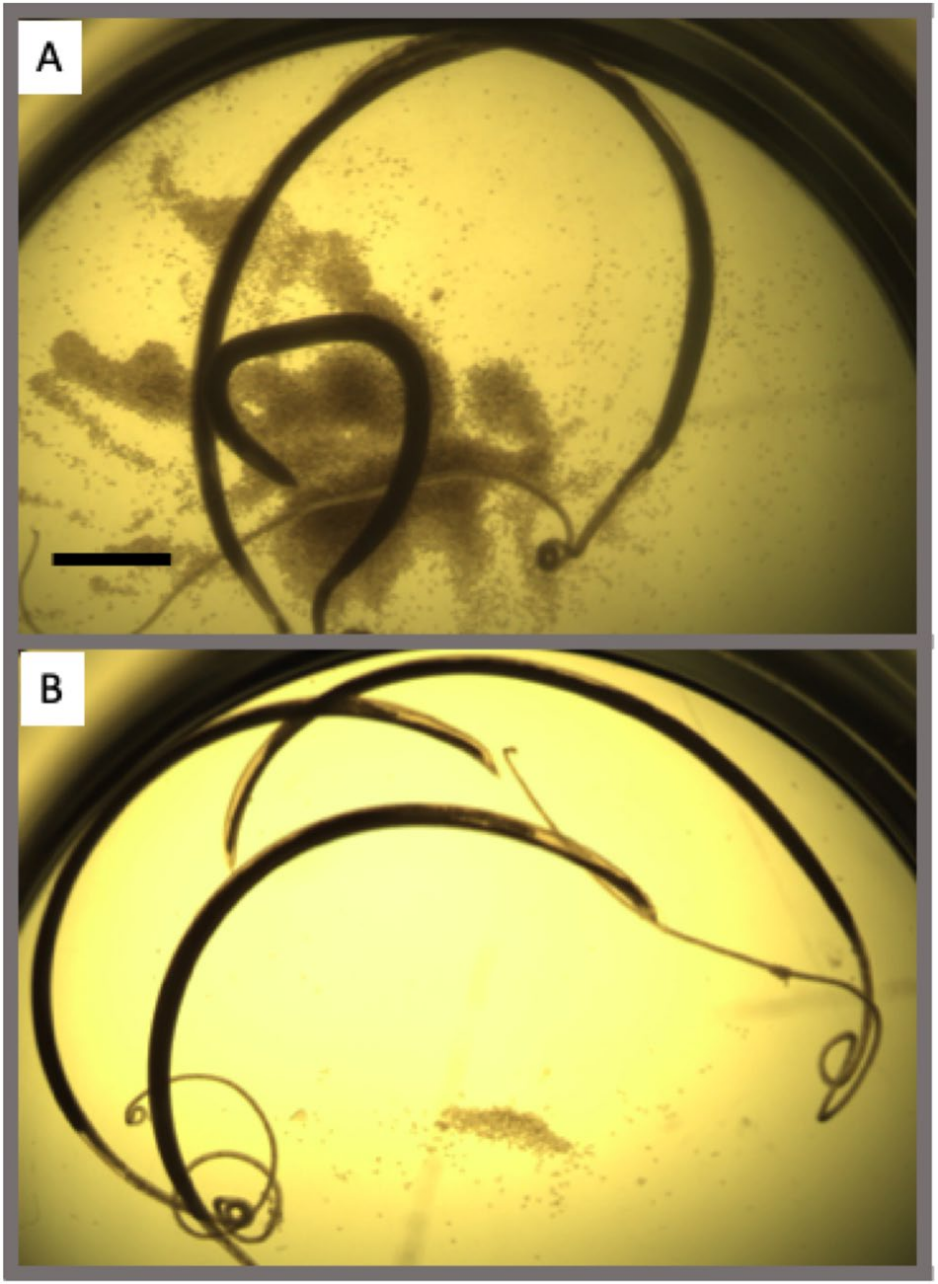
Adult *T*. *muris* whipworm example screening in 24 well plate. (**A**) DMSO healthy and motile whipworms, with an abundance of released eggs; (**B**) Dead whipworms in well with active compound.

As a second independent measure of positives’ quality and for further prioritization of compounds, we performed a secondary *in vitro* screen of the adult hookworm actives against the adult stage of a distantly related intestinal nematode parasite, whipworm. Drugs that target multiple STNs are of much higher priority than drugs that target only individual parasites—*e*.*g*., due to the economics of drug development and the practicality of MDA in resource poor populations. *T*. *muris* whipworm adults were screened *in vitro* against as many of the adult hookworm standard positives as we could readily get access to from commercial suppliers (32 out of 39). Out of those 32 drugs, 19 were found to be effective against whipworms at a 100 µM dose (Fig. 4; Tables 2, S1). Moreover, the hookworm E2L screening was again superior to any of the *C*. *elegаns* screens in recalling compounds with broad-spectrum activity against adult nematode parasites. Hookworm E2L was able to detect 16 of the 19 actives against *T*. *muris*, compared to only eight and six detected using *C*. *elegans* L4 and E2A, respectively (Table 2). All of the whipworm adult positives captured by *C*. *elegans* were also captured by hookworm E2L.

**Table 2:** The total number of *T*. *muris* actives out of 32 drugs active against *A*. *ceylanicum* adults

	*T*. *muris* adults 100 µM	*A*. *ceylanicum* E2L 30 µM	*C*. *elegans* L4 30 µM	*C*. *elegans* E2A 30 µM
Total Actives	19	21	12	6
Whipworms overlapping actives	.	16	8	6
Whipworms missed actives	.	3	11	13

. Each model tested was reevaluated for its ability to detect those drugs that affect whipworms adults. Out of the 32 hookworm standard actives, 19 showed activity against whipworms. Among all model tested E2L showed the highest number of overlapping actives (16 out of 19) with adult whipworms, compared to 8 and 6 in *C*. *elegans* L4 and E2A.

The broad-spectrum STN adult actives recalled by hookworm E2L, but not by the *C*. *elegans* screens, also tend to display lower cytotoxicity profile (Table S3), with only one out of seven such hits (positive on hookworm E2L but negative on *C*. *elegans*) exhibiting submicromolar activity in the PubChem cytotoxicity assays discussed above (AID743012 or AID1224886). On the other hand four out of nine broad-spectrum STN adult actives recalled by the *C*. *elegans* L4 assay show cytotoxicities in the submicromolar range in the same assays. In addition, seven out of nine *C*. *elegans*-recalled actives have legacy murine oral LD50 values in the high toxicity range (<300 mg/kg), whereas only two out of seven broad spectrum actives recalled only by the hookworm E2L assay were in this range, highlighting the potential value of parasitic E2L as a primary screening system over *C*. *elegans*.

### *In vivo* screening against *A. ceylanicum*

**Figure 5:**
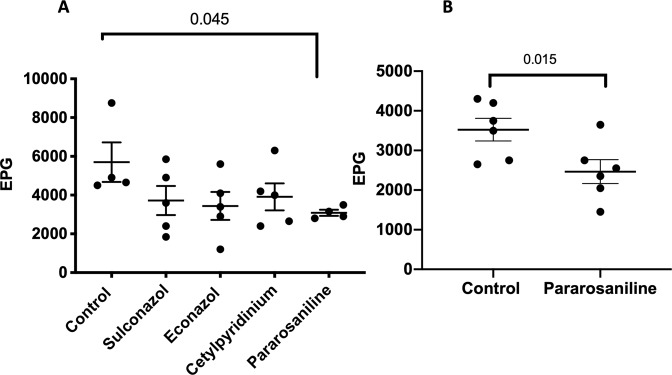
*In vivo* activity of down-selected library compounds against *A*. *ceylanicum* infections in hamsters. (**A**) Eggs per gram of feces (EPG) in hamsters given a single oral dose of 50 mg/kg of each of the four compounds compared to water control. Only pararosaniline showed significant reduction (46%) compared to control (all other comparisons relative to control did not reach significance); (**B**) EPGs in hamsters given a single oral dose of 100 mg/kg pararosaniline, showing significant reduction (30%) relative to control. Numbers above bars indicate P values. Long horizontal lines represent EPG means; short horizontal lines represent standard error.

Of the 19 broad-spectrum STN adult actives, four were prioritized and selected for *in vivo* testing. These four were selected on the basis of their putative and inferred targets in parasites, the importance of these targets to parasitism, and in the safety profile of these drugs. The four drugs selected were econazole nitrate, sulconazole nitrate, pararosaniline hydrochloride, and cetylpyridinium chloride. In prior repurposing screens, the first two drugs were found to have potent inhibitory activities against a cytochrome P450 (CYP-450) in a parasitic flatworm^46^ whereas the latter two were found to be effective against an amoeboid parasite by targeting its Heat Shock Protein 90 (HSP-90)^47^. Both of these are believed to play critical roles in both parasite viability and the process of parasitism^48–50^. We screened these four drugs directly in hamsters infected with *A*. *ceylanicum*. Among all four treatments, one and only compound, pararosaniline, showed significant reduction in fecal egg counts (Fig. 5A; no significant reduction in worm burdens seen; Fig. S1A). This result was independently repeated and pararosaniline was re-tested against *A*. *ceylanicum*-infected hamsters (Figs 5B, S1B).

## Discussion

In this work we have compared the efficiency of different screening models for STN drug discovery. We considered adult *in vitro* screening as the best standard, since it is the life stage responsible for disease burden in vertebrate hosts, then evaluated each model for its ability to detect hits that also affect adults (true positive rates or TPRs), as well as false positive and false negative rates. Considering the dose of 30 µM, which was used for all stages screened, hookworm E2L screen was associated with the highest TPR with the adult hookworm actives (27/39) and the lowest number of the corresponding false negatives (12). On the other hand, *C*. *elegans* displayed a far worse TPR (11/39 for E2A and 14/39 for L4) and a significantly higher number of false negatives (28 for E2A, 25 for L4). Based on our data, *C*. *elegans* will therefore miss at more than twice as many adulticidal hits as parasitic larvae. L3i and xL3i were also inferior to hookworm E2L with regards to TPR. With regards to known anthelmintics, we found 15 in the library with known activity against one or more nematodes. Of these, hookworm E2L correctly identified 13/15. The only two it did not find were morantel and oxantel, two compounds with narrow spectrum of activity that are known to not work well against hookworms^36,51^ (these were both missed by *C*. *elegans* as well), further validating the usefulness of hookworm E2L for drug screening.

Although hookworm E2L also resulted in a higher number of false positives, false positives in a primary screen of a multi-step screening campaign is less critical than the false negatives since the later represents a permanent dismissal of a potentially the best candidates and since a high number of false positives in a primary screen could be trimmed effectively by secondary screens (*e*.*g*., against parasite adults). Moreover, many compounds that we termed “false positives” were structurally similar to their “true positive” counterparts and, likely, just didn’t reach the established activity threshold. For example many other azoles structurally related to econazole and sulcanazole (miconazole and oxyconazole) were not as effective against hookworm adults, but were sufficiently active against the E2L system to be registered as hits. These “false positives” do then bring an added value of validating both the “true positive” hits and their putative targets. One caveat to our findings is that our conclusions are based upon screening of a library that contained 1280 compounds. The screening of this library to its entirety or near entirety against adult parasites, larval parasitic stages, and two *C*. *elegans* stages is unprecedented and provides a solid foundation for comparison.

Other analyses and data support these conclusions. First, parasite-specific positives show lower overall toxicity than positives that overlap between the parasites and *C*. *elegans*. Furthermore, hookworm larvae are much more likely to discover positives that have broader spectrum against STN adults (hookworms + whipworms) than *C*. *elegans*. Again, such positives are less likely to be generally toxic. Taken together, our results indicate that, whereas *C*. *elegans* is capable of detecting some anti-STN drugs, it is much more likely to miss the best candidates in terms of adult parasitic activity and lower toxicity. These findings were confirmed by *in vivo* screening of our prioritized candidates. Of the four candidates tested *in vivo*, the only one that showed significant impact against the parasites was picked up by hookworm E2L but was absent in all *C*. *elegans* screens (and hookworm L3i and xL3i). As expected for a non-optimized hit from a small library, pararosaniline is not as effective against hookworms as well-known fully developed anthelmintics that have been used clinically for decades, such as albendazole, pyrantel, and ivermectin, all of which have strong effects *in vivo* at 6 mg/kg in this model^52^.

Consistent with our findings, it has been noted that, although the use of *C*. *elegans* as a model for parasitic nematodes started 30 years ago with tens of millions of drugs having been screened against it, no single compound discovered with *C*. *elegans* has yet reached the clinic^31^. Indeed, animal health pharmaceutical laboratories have been the source of all the anthelmintics in use now, including against human STNs. For example, three of the latest drugs in use against gastrointestinal nematodes, paraherquamide, emodepside, and monepantel, were found in screens against parasitic nematodes of veterinary importance, although little or no details of how these screens were carried out were given^53–55^. Interestingly, in our research on the initial discovery of deworming drugs used today^35,53,54,56–59^, five were discovered as anthelmintics screening *in vivo* against parasitic nematode infections in whole host-animals (ivermectin, paraherquamide, emodepside, levamisole, and pyrantel) and two were discovered using parasitic larval assays (benzimidazoles, monepantel), further validating the importance of parasite larvae as an effective screening tool, in this case for human STNs.

Our screening results also highlight two important putative targets for STN drug development, CYP-450 and HSP-90. *C*. *elegans* harbors a large repertoire of >60 cytochrome P-450 genes^60^, a phenomenon that is believed to enhance drugs biotransformation and elimination, perhaps contributing to *C*. *elegans* as a poor model for phenotypic anthelmintic screens. In contrast, parasitic nematodes harbor fewer CYP-450 enzymes. In fact, it was once believed that CYP-450 activity was generally absent in parasitic nematodes^61^. However, recent studies have shown that parasitic nematodes possess CYP-450 enzymes, albeit in limited quantity, and established their essential roles in the process of parasitism^48^. CYP450’s are heme-containing monooxygenases that play critical roles in xenobiotic biotransformations and in the metabolism of essential endobiotic compounds such as sterol, cholesterol and ergosterol^62–64^. The imidazole derivatives-econazole and sulconazole- were developed mainly as antifungal drugs inhibiting fungal CYP450’s, leading to a decreased production of ergosterol, the essential bioregulator of fungal cell membrane fluidity and integrity^65–67^. The CYP-450 inhibitors, econazole and sulconazole, were found to display antiparasitic activity against *Schistosoma mansoni*^46^, antitrypanosomal activity^68^ against *Trypanosoma cruzi*, and antibacterial activity against *Mycobacterium tuberculosis*^69^ and against the drug resistant *Salmonella typhimurium*^70^. The antiparasitic activity of imidazoles is believed to act through their inhibitory activity against CYP-450’s that was recently found to be essential for the survival of protozoan and helminth parasites^46,50^ and linked to the anthelmintic drug resistance^71^.

Similarly, HSP90 is an essential enzyme for some parasites and is believed to act as a sensor in the process of transition between different hosts and environments-with dramatic change in temperature, pH and exposure to immunological insults-during development, such transition require prompted adaptation^72^. HSP-90 is an evolutionary conserved chaperon protein essential in all eukaryotes, that is responsible for maintaining organisms in homoeostasis and may act as a guard for client proteins against host insults^73^. Pararosaniline and cetylpyridinium were previously reported to have inhibitory activities against parasite HSP-90s and to interrupt the development and growth of protozoan parasites, including *Entamoeba histolytica*^47^ and *Plasmodium falciparum*^74^. Pararosaniline was also years ago subjected to a field study for the anthelmintic activity against schistosomiasis^75–77^. In filarial parasitic nematodes, HSP-90 is an essential enzyme and is the subject of drug screens^78–80^. Our data suggest that both HSP-90 and CYP-450 are potentially good targets for controlling human STNs.

Our interesting finding that the free-living stage of hookworm is a much better predictor of positive drug activity against adult hookworms than the free-living *C*. *elegans* has potentially interesting implications for evolution and parasitism. The free-living stages of hookworm larvae and *C*. *elegans* have many similarities, living in the environment and feeding on bacteria. Therefore, a priori, one might think that free-living larval hookworm stages have more in common with the free-living *C*. *elegans* than with the adult parasite. We speculate that the environmental stages of hookworms and *C*. *elegans* are more different than might appear on the surface, as has been noted by others in the context of dauer vs L3i development^81,82^. Hookworm larvae only need to exist as free-living worms for a few days before entering the infectious larval stage, awaiting a human host for entry, where it will complete its life cycle and reproduce, living for years. Thus, the vast majority of its life (and all of its reproductive cycle) is spent in the host. Conversely the entire life and reproductive cycles for *C*. *elegans* are spent in the environment. Therefore, we speculate that whereas *C*. *elegans* has evolved a genome to deal with major environmental stresses for its entire lifespan, hookworms, which evolved to parasitize a long time ago^83^, have evolved so that their genomes are primarily adapted to long survival in the host. Consequently, the genes retained by hookworms for coping with external environmental threats (such as genes encoding xenobiotic drug transporters) may be at least somewhat atrophied by comparison with *C*. *elegans* (so that testing anthelmintics on *C*. *elegans* gives negative results that would not be observed in actual hookworms). Along these speculative lines, one might imagine that the great expansion of GPCR genes in *C*. *elegans* relative to parasites like hookworms^84,85^ reflects a greater need to deal with changing environmental conditions versus a relatively more constant condition in the human small intestine, and that the smaller repertoire of GPCRs required for parasitism are thus used by the parasite in the short free-living stage as well. These speculations could be tested, for example, by seeing which GPCRs are expressed in both developing hookworm larvae and adult hookworms and comparing these to GPCRs expressed in *C*. *elegans* larvae at similar times as the hookworm larvae. These speculations could also indicate why hookworm larvae have a higher number of false positives—developing hookworm larvae might depend on a smaller, less redundant set of genes for survival in the environment than *C*. *elegans*, which must spend its whole life there.

**Figure 6:**
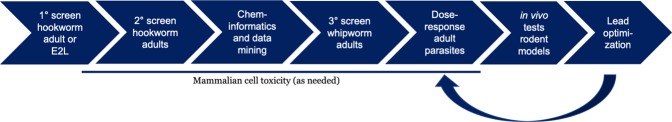
Proposed drug screening pipeline based on results shown here. When the screening library is small enough (*e*.*g*., <2000 compounds) we propose screening directly against adult hookworm parasites to maximize quality actives. For larger libraries, we propose screening against hookworm E2L followed by secondary screening against adult hookworm parasites. Cheminformatics and data mining can then be employed to further downselect. Hookworms adults active compounds will be further prioritized by screening against adult whipworms at a single dose and then, if necessary, using a dose response against the adult stages of both parasites to select for potent and broad-spectrum actives prior to *in vivo* testing. Mammalian cell screening can be employed at any point to eliminate compounds that may be overly toxic.

Based on our results, we propose a screening pipeline focused on a parasitic nematodes as the main screening model (Fig. 6). The decision of choosing between these models could be dependent on the size of a screening library. For example, if a screening library is <2,000 compounds, then it might be reasonable to screen directly against adult hookworm parasites; however, if the screening library is >10,000 compounds, then hookworm E2L will be the most efficient screening model. Hits from such a primary screen will be subjected to data mining, cheminformatic analysis, and, possibly, if not known, a mammalian cell toxicity screen, to down select superior candidates on the basis of an existing chemotype record, its predicted potential for drug development, the essentiality of the putative target, and safety profile. Compounds that pass these filters will go through a secondary screen and validation against hookworm adult parasites (if initially screened against E2L) and/or whipworm adult parasites. Compounds with broad spectrum anthelmintic activity could be subjected to further down-selection based on data mining/chemoinformatics and dose responses against both adult parasites followed by *in vivo* tests using STN-infected laboratory animals.

## Methods

### Maintenance of parasites and *C. elegans*

*A*. *ceylanicum* parasites were maintained in golden Syrian hamsters following standard protocols^86–89^. *Trichuris muris* parasites were maintained in STAT6−/− mice^52^. *C*. *elegans* wild type strain N2 Bristol was maintained as per standard protocols^90^.

### Drugs and library

A MicroSource library of 1280 compounds (FDA-approved US drug collection of 1040 compounds and International drug collection of 240 compounds; Supplemental File 1) was obtained from the Small Molecule Screening Facility, University of Massachusetts Medical School Worcester. Screening library was obtained in 96-well plate format and a concentration of 1 mM and 20% DMSO. Selected hits from the screening library for *T*. *muris in vitro* screening were obtained from Milipore Sigma (Sigma Aldrich) and Toronto Research Chemicals.

### Adult hookworm *in vitro* screening

Young adult *A*. *ceylanicum* were harvested from the small intestine of infected hamsters day 11 post-inoculation (PI) and washed three times using prewarmed medium (RPMI1640, 100U penicillin, 100 µg/ml streptomycin, 10 µg/ml amphotericin). Three worms were manually picked into each well of the 96-well screening plate, containing 97 µl of medium. Using a multichannel pipette 3 µl of 1 mM (20% DMSO) drug plates were dispensed into screening plates giving a final concentration of 30 µM (0.75% DMSO). In our assays, we left out serum to avoid any possible drug binding and/or metabolism with serum proteins and/or enzymes. As such, assays have to be scored in a shorter than normal period of time as the health of control hookworm adults degenerates more quickly in the absence of serum. Nonetheless, the adults stayed very healthy throughout the duration of the assay and the scheme requires that the drugs act quickly (which we viewed as a robust positive facet due to potentially limited exposure time of intestinal nematode parasites to drugs as the drugs transit through the intestinal tract). Assay plates were incubated for 24 hours at 37 °C and 5% CO_2_. Drug activity was determined using the standard motility index^52^. Motility index of 3 were given to vigorous worms, 2 for motile worms, 1 for motile after stimulation by touching and 0 for dead worms. Drug was considered effective or a positive, if at least two worms have motility index of ≤1. To validate the results, from two library plates (one with a high number of actives and one with a low number of actives) representing ~12% of the total library were rescreened on adults. Results from re-screen was identical with the initial screen.

### Hookworm developmental screening (E2L)

For the egg-to-larva (E2L) hookworm development screen, about 120 third-staged infectious larvae (L3i) were orally gavaged into male Syrian hamsters immunosuppressed with dexamethasone 1 mg/L in drinking water. Immunosuppressed hamsters can give a significant yield of eggs for up to 70 days compared to 12 days in immunocompromised animals. Fecal material was collected starting 18 days post infection and about 20 grams of feces were processed for egg isolation and purification following salt/sucrose flotation protocol^91^. Using a multichannel pipette, About 40–60 eggs were dispensed into each well of 96-well plate containing *E. coli*, OP50, as source of food with a final OD600 of 0.3, and a final test drug concentration of 10 µM or 30 µM in 0.25% or 0.75% DMSO respectively in 100ul *C*. *elegans* S medium^92^. Test plates were incubated at 28 °C for 7 days then scored for development. Test drug was considered hit if >~90% of the eggs as judged by eye failed to hatch or hatched but failed to develop into L3i.

### *C. elegans* E2A screening

Small agar pieces of recently starved plates of *C*. *elegans* were “chunked” unto Enhanced Nematode Growth (ENG) plates^93^ spread with *E*. *coli* OP50 and incubated at 20 °C for 48 hours. Eggs were prepared by bleaching the gravid adults, which were then washed thrice to remove bleach and re-suspended in S medium. About 50–60 eggs were dispensed into each well of 96-well plates containing, *E*. *coli*-OP50 (OD600 of 0.3) and a final drug concentration of either 10 µM or 30 µM (0.25% or 0.75% DMSO respectively) and a final volume of 100 µL. Plates were wrapped in moist towels to keep hydrated and incubated at 25 °C for 72 hours, at which point >90% of controls have developed to early adult stage. Test drug were considered active if >90% of the eggs as judged by eye either failed to hatch or hatched but failed to develop into adults.

### *C. elegans* L4-adult screening

Synchronized population of L1 larvae were prepared from bleached eggs (see above) after allowing them to hatch overnight in M9 medium^94^ at room temperature. First-staged larvae (L1s) were plated on ENG plates seeded with *E*. *coli*-OP50 then incubated at 20 °C for 44 hours until the fourth larval (L4) stage. After incubation, L4-worms were washed of the plates using M9 medium and washed trice. About 20 L4-worms were dispensed into each well of a 96-well plate containing *E*. *coli* OP50 (OD600 of 0.3), S medium, and 30 µM of test drugs, with a final concentration of 0.75% DMSO and final volume of 100 µL. Plates were incubated at 25 °C for 96 hours. Drug were considered effective against *C*. *elegans* if they killed and/or paralyzed >~80% of worms as judged by eye or gave rise to no/very low brood sizes (sterilization of progeny production).

### Hookworm L3i and xL3i screening

L3i from standard cultures were washed trice using S medium and tested in the drug screen. For screens with exsheathed L3i^43^, L3i were incubated in saline containing 0.2% sodium hypochlorite for about 6 min, until full exsheathment was observed under the light microscope^95^. Exsheathed L3i (xL3i) were incubated in LB medium supplemented with 100 U/ml of Penicillin, 100 µg/ml Streptomycin and 2.5 µg/ml amphotericin B)^43^. Half of the library plates (640 drugs) were screened against L3i in S medium and xL3i in LB medium (final volume, 100 µL for each). About 50–60 L3i or xL3 were transferred into each well of the 96-well plate containing 30 µM of test drugs and a final concentration of 0.75% DMSO, and then incubated for 72 hours at room atmosphere and temperature for L3i or at 37 °C and 10% CO_2_ for LB induced xL3i. Test drug was considered hit if it >~90% as judged by eye of the infective larvae showed no motility.

### *In silico* analysis of screening hits

Cheminformatics on screening hits and analysis of legacy data were carried out using Vortex (Dotmatics, Inc.) and the online PubChem database (https://pubchem.ncbi.nlm.nih.gov/) was used to mine data. The list of hits was assembled via the PubChem database, and the SD structure file was downloaded and processed via Vortex. Chemical fingerprints (DotFPCA) were generated and co-clustered using the K-means Clustering tool to identify any common chemotypes. The PubChem BioActivity Summary tool (no longer supported for multiple-compound input after Nov. 1, 2018) was employed to identify deposited PubChem assays that provided dose-dependent cytotoxicity data on the largest number of the anthelmintic candidates from the FDA-approved drug library.

### Adult whipworm screening

*T*. *muris* adult whipworms were harvested from the cecum and small intestines of infected STAT6 −/− mice. Three worms were manually picked into each well of a 24-well plate, 2 wells/ test drug and a final volume of 1 ml of medium (as for hookworm adults above). Out of the 39 drugs that hit adult hookworms, 32 that were readily available for repurchase were tested against whipworms at final concentrations of 100 µM (0.75% DMSO), a dose based on the efficacy of known anthelmintics against *T*. *muris* adults *in vitro* and based on previous drug screens^36,52,96^. Pararosaniline pamoate was unavailable from commercial sources instead pararosaniline hydrochloride was purchased and tested against whipworm adults and the following *in vivo* experiments. Test plates were incubated for 48 hours at 37 °C and 5% CO_2_. Drug activity was assayed using the standard motility index as per hookworm adults. Test drug was considered effective against whipworms if at least 4 of the 6 tested worms showed motility index of ≤1. DMSO-only controls stayed healthy during the duration of the test.

### Hookworm *in vivo* study

Four to five-week old male hamsters were infected with ~120 *A*. *ceylanicum* L3i. On day 17 post inoculation, the number of eggs/gram feces was counted using a modified McMaster technique^97^. Infected animals were grouped accordingly into four similar treatment groups and a placebo control. Animals in the treatment groups were orally treated with 400 ul dH_2_O containing 50 mg/kg of either sulconazole nitrate, econazole nitrate, cetylpyridinium chloride or pararosaniline hydrochloride. Two days post treatment, hamsters were subjected to an overnight fecal collection. Three days post treatment, animals were euthanized and small and large intestines were harvested for worm burden assessment^52,89^. For the repeat experiment, 100 mg/kg pararosaniline was used. Data analysis of intestinal worm burden and fecal egg counts was plotted using Prizm 8 (GraphPad software Inc, La Jolla, CA, USA). Dots represent the individual worm burdens or fecal egg counts for each hamster in each treatment group. In the first *in viv*o test, SPSS v. 25 Dunnett’s many-to-one comparison comparing each treatment to the untreated control. In the second *in vivo* test with pararosaniline statistical analysis was done using Prizm 8, one-tailed Mann-Whitney U test.

### Ethical approval

All animal experiments were carried out under protocols approved by the Institutional Animal Care and Use Committee (IACUC) of the University of Massachusetts Medical School (UMMS A-2483 and UMMS A-2484). Hamsters and mice used in this study were housed, handled, fed and experimentally used following the National Institute of Health (NIH) Guide for the Care and Use of Laboratory Animals in Research (see 18-F22). Euthanasia was performed by CO_2_ asphyxiation, followed by bilateral pneumothorax.

## Supplementary information

Supplementary Data
