# Supplementary material for: Drug Screening for Discovery of Broad-spectrum Agents for Soil-transmitted Nematodes
Source: Sci Rep. 2019 Aug 26;9:12347. doi: 10.1038/s41598-019-48720-1 (PMC6710243; doi:10.1038/s41598-019-48720-1)
Supplement: Supplementary file 1 — Supplementary Data [file 41598_2019_48720_MOESM1_ESM.docx]

**Supplementary data for:**

**Drug Screening for Discovery of Broad-spectrum Agents for Soil-transmitted Nematodes**

Mostafa A. Elfawal^1^, Sergey N. Savinov^2^, and Raffi V. Aroian^1*^

^1^Program in Molecular Medicine, University of Massachusetts Medical School Worcester

^2^Department of Biochemistry and Molecular Biology, University of Massachusetts Amherst

| MOLENAME | HW Adult 30 µM | HW E2L 10 µM | HW E2L 30 µM | *C. elegans* L4 30 µM | *C. elegans* E2A 10 µM | *C. elegans*  E2A 30 µM | HW  iL3  30 µM | HW LB_xiL3  30 µM LB | WW 100 µM |
| --- | --- | --- | --- | --- | --- | --- | --- | --- | --- |
| PHENAZOPYRIDINE HYDROCHLORIDE | ***** | ***** | ***** | ***** |  |  | ***** | ***** |  |
| FENBENDAZOLE | ***** | ***** | ***** | ***** |  |  | ***** | ***** |  |
| FEBUXOSTAT | ***** |  |  |  |  |  |  |  |  |
| SULCONAZOLE NITRATE | ***** |  | ***** | ***** |  | ***** |  |  | ***** |
| RONIDAZOLE | ***** |  |  |  |  |  |  |  |  |
| ECONAZOLE NITRATE | ***** | ***** | ***** |  |  |  |  |  | ***** |
| PENFLURIDOL | ***** | ***** | ***** | ***** | ***** |  | ***** | ***** | ***** |
| DICHLORVOS | ***** |  | ***** |  |  |  | ***** | ***** | ***** |
| IVERMECTIN | ***** | ***** | ***** | ***** | ***** | ***** | ***** |  | ***** |
| BISACODYL | ***** |  |  |  |  |  |  |  |  |
| CETYLPYRIDINIUM CHLORIDE | ***** | ***** | ***** | ***** |  | ***** |  | ***** | ***** |
| GENTIAN VIOLET | ***** | ***** | ***** | ***** |  | ***** |  |  | ***** |
| HEXACHLOROPHENE | ***** |  | ***** |  |  |  |  |  | ***** |
| PHYSOSTIGMINE SALICYLATE | ***** |  |  |  |  |  |  |  |  |
| PYRANTEL PAMOATE | ***** |  | ***** |  |  |  |  |  | ***** |
| THIABENDAZOLE | ***** | ***** | ***** |  |  |  | ***** | ***** |  |
| THIMEROSAL | ***** | ***** | ***** | ***** | ***** | ***** |  | ***** | ***** |
| PHENYLMERCURIC ACETATE | ***** | ***** | ***** | ***** | ***** | ***** | ***** |  | ***** |
| MONENSIN SODIUM | ***** |  |  |  |  |  |  |  | ***** |
| QUINAPRIL HYDROCHLORIDE | ***** |  |  |  |  |  |  |  |  |
| PARAROSANILINE PAMOATE | ***** | ***** | ***** |  |  |  |  |  | ***** |
| PERHEXILINE MALEATE | ***** |  | ***** |  |  |  |  |  | ***** |
| LEVAMISOLE HYDROCHLORIDE | ***** | ***** | ***** | ***** | ***** |  |  |  | ***** |
| OXIBENDAZOLE | ***** | ***** | ***** | ***** |  |  |  |  |  |
| NIMODIPINE | ***** |  |  |  |  |  |  |  |  |
| BENZALKONIUM CHLORIDE | ***** | ***** | ***** |  |  |  |  |  | ***** |
| MOXIFLOXACIN HYDROCHLORIDE | ***** |  |  |  |  |  |  |  |  |
| OXFENDAZOLE | ***** |  | ***** | ***** |  |  |  |  |  |
| PROTRYPTYLINE HYDROCHLORIDE | ***** |  |  |  |  |  |  |  | ***** |
| NICOTINE DITARTRATE | ***** |  |  |  |  |  |  |  | ***** |
| CHINIOFON | ***** |  |  |  |  |  |  |  |  |
| HEXETIDINE | ***** |  | ***** |  |  |  |  |  | ***** |
| **SALINOMYCIN, SODIUM** | ***** |  | ***** |  |  |  |  |  |  |
| **ALEXIDINE HYDROCHLORIDE** | ***** | ***** | ***** |  |  | ***** |  |  |  |
| **CETRIMONIUM BROMIDE** | ***** | ***** | ***** |  | ***** | ***** |  |  |  |
| **MORANTEL CITRATE** | ***** |  |  |  |  |  |  |  |  |
| **SELAMECTIN** | ***** | ***** | ***** | ***** | ***** | ***** |  |  |  |
| **BENZETHONIUM CHLORIDE** | ***** |  | ***** |  |  |  |  |  |  |
| **PYRVINIUM PAMOATE** | ***** | ***** | ***** | ***** |  | ***** |  |  |  |

Table-S1 List of the 39 compounds active against *A. ceylanicum* adult hookworms (HW) and their cross-activity against all other nematode and nematode stages screened. Among those, 32 were tested against *T. muris* whipworm (WW) adults. * indicates compound was active against nematode. Shaded cells indicate compound was not tested against nematode.

Table-S2. Mined cytotoxicity data for the adult hookworm actives.

| **Compound Name** | **PubChem CID^a^** | **Selectivity Class^b^** | **DT40 IC50, uM (PubChem AID743012)^c^** | **DT40 Cytotox. Ranking^d^** | **HEK293 IC50, uM (PubChem AID1224886)^e,f^** | **HEK293 Cytotox. Ranking^d^** |
| --- | --- | --- | --- | --- | --- | --- |
| PYRVINIUM PAMOATE | 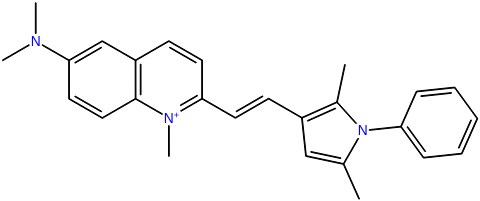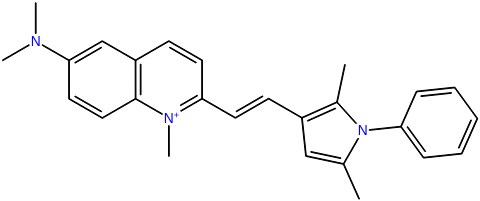54680693 | NG | 0.25 | High | NT |  |
| PHENYLMERCURIC ACETATE | 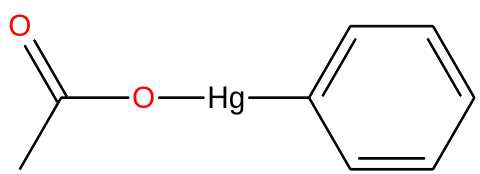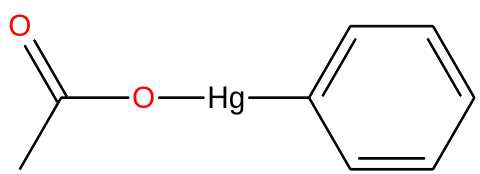16682730 | NG | 0.11 | High | 0.24 | High |
| GENTIAN VIOLET | 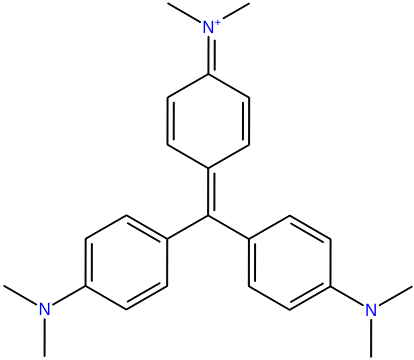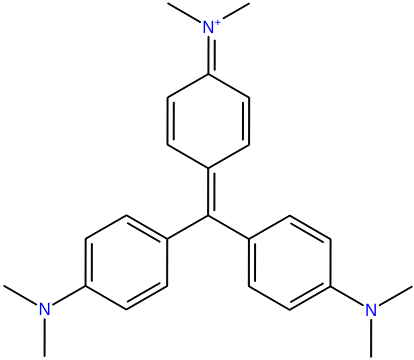11057 | NG | 0.19 | High | 5.47 | Moderate |
| THIMEROSAL | 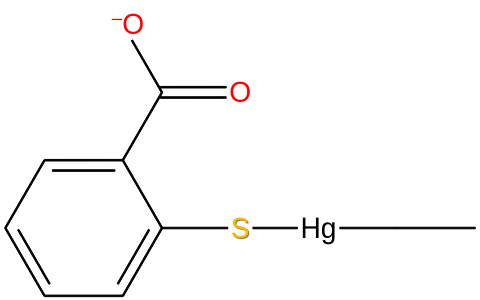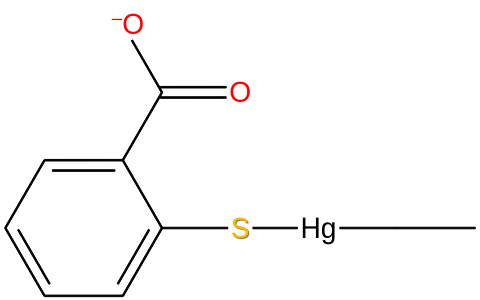16684434 | NG | 0.17 | High | 1.10 | Moderate |
| PENFLURIDOL | 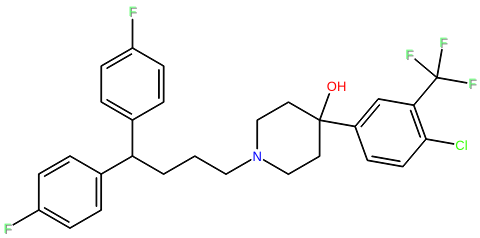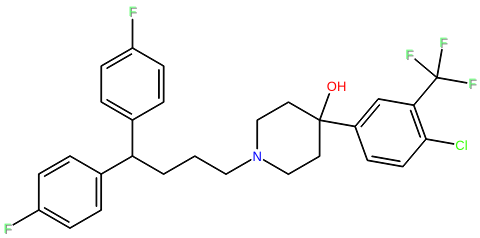33630 | NG | 4.22 | Moderate | 9.52 | Moderate |
| CETYLPYRIDINIUM CHLORIDE | 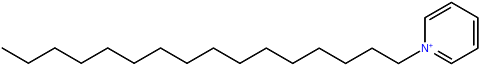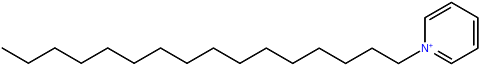2683, 31239 | NG | 0.43 | High | 17.37 | Low |
| SULCONAZOLE NITRATE | 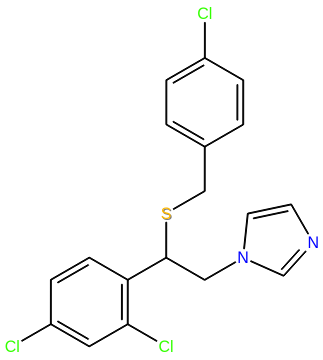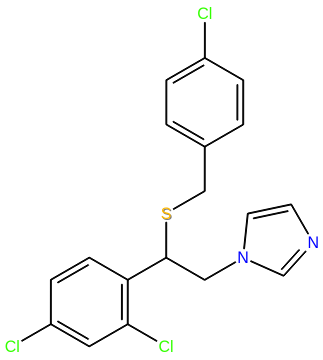5318, 65495 | NG | 10.12 | Low | 16.93 | Low |
| PHENAZOPYRIDINE HYDROCHLORIDE | 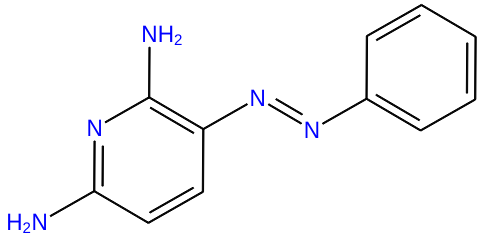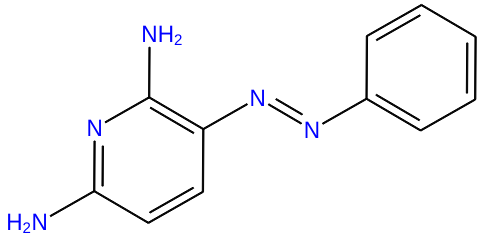4756, 8691 | NG | 4.22 | Moderate | 24.54 | Low |
| IVERMECTIN | 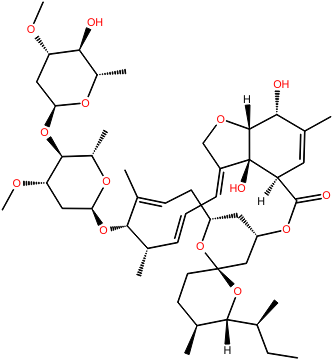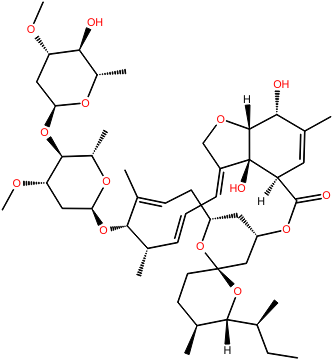3085416, 11957587 | NG | NT |  | NT |  |
| SELAMECTIN | 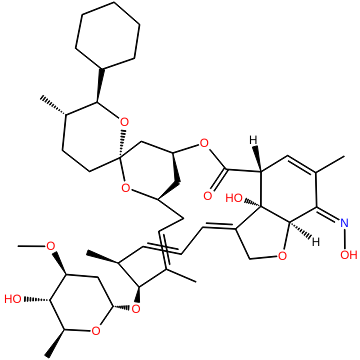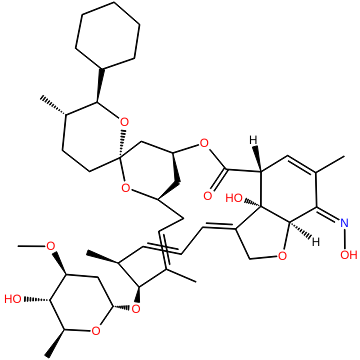9578507 | NG | Inactive | Low | NT |  |
| OXFENDAZOLE | 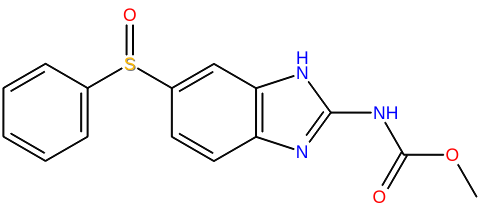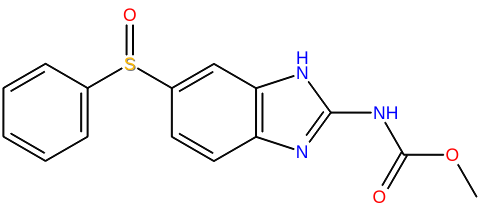40854 | NG | 9.44 | Moderate | 0.95 | High |
| OXIBENDAZOLE | 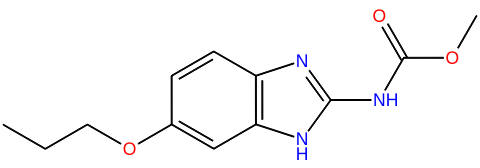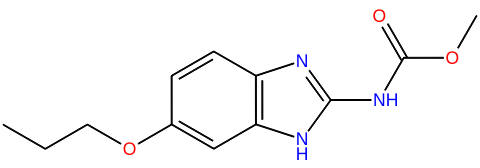4622 | NG | 0.23 | High | 0.17 | High |
| FENBENDAZOLE | 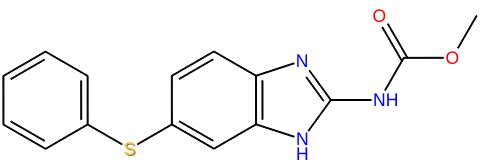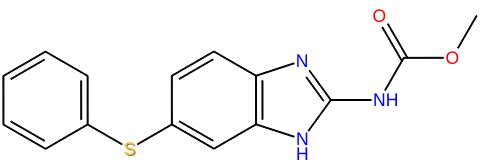3334 | NG | 0.40 | High | 0.87 | High |
| LEVAMISOLE HYDROCHLORIDE | 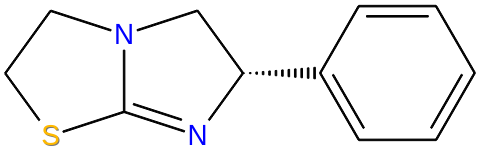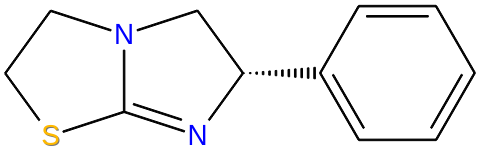26879, 27944 | NG | Inactive | Low | Inactive | Low |
| BENZALKONIUM CHLORIDE | 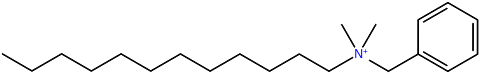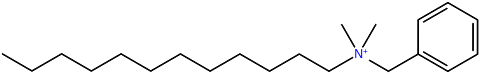23705 | PS(AD) | 0.42 | High | 3.01 | Moderate |
| MONENSIN SODIUM | 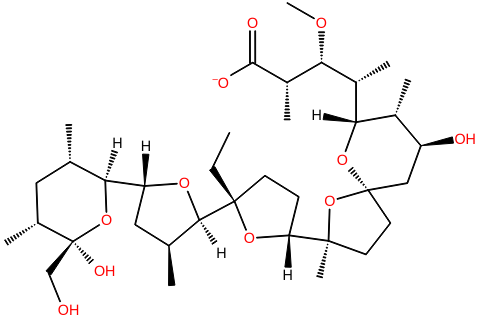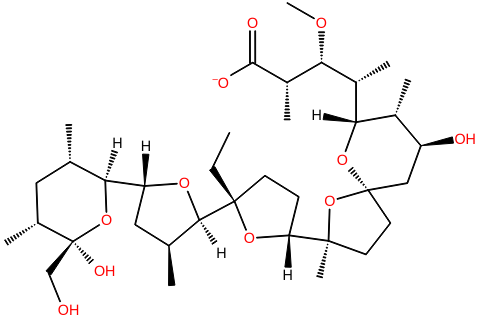441145. 23667299 | PS(AS) | 0.47 | High | NT |  |
| FEBUXOSTAT | 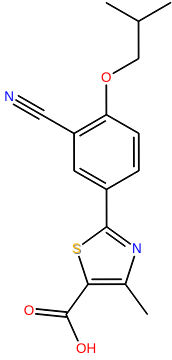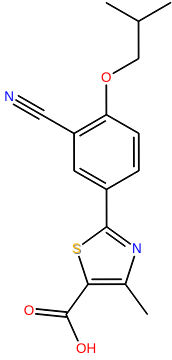134018 | PS(AS) | Inactive | Low | Inactive | Low |
| HEXACHLOROPHENE | 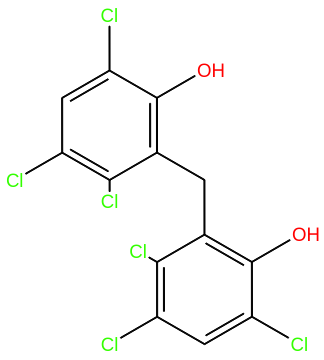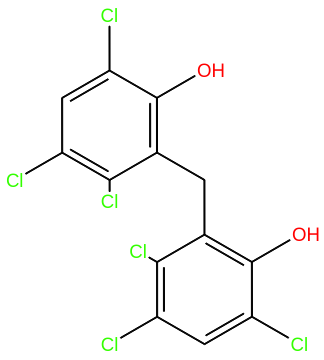3598 | PS(AS) | 3.05 | Moderate | 3.88 | Moderate |
| ALEXIDINE HYDROCHLORIDE | 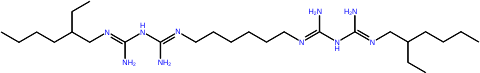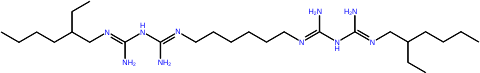2090, 102678 | PS(AD) | 8.41 | Moderate | 5.35 | Moderate |
| CETRIMONIUM BROMIDE | 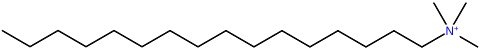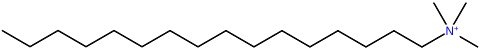5974, 8154 | PS(AD) | 0.47 | High | 9.42 | Moderate |
| PARAROSANILINE PAMOATE | 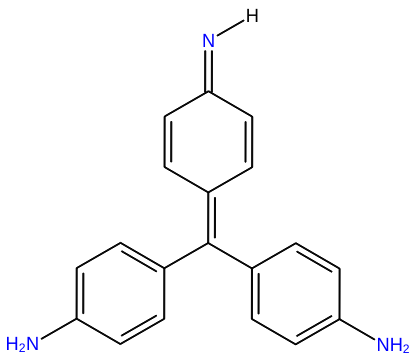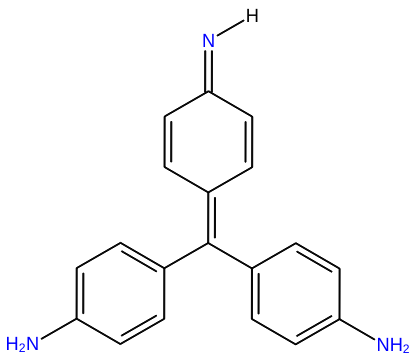11292, 11293 | PS(AD) | 9.69 | Moderate | 3.47 | Moderate |
| ECONAZOLE NITRATE | 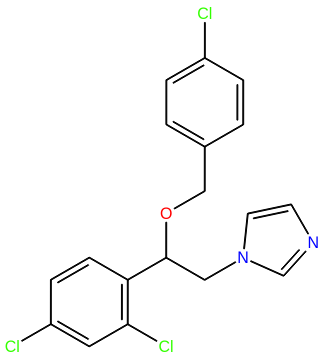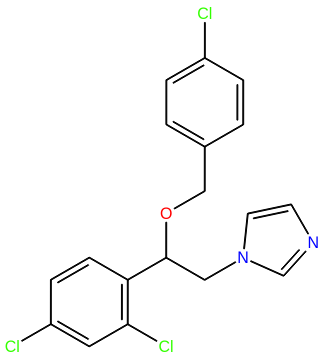3198, 68589 | PS(AD) | 7.50 | Moderate | 21.79 | Low |
| CHINIOFON | 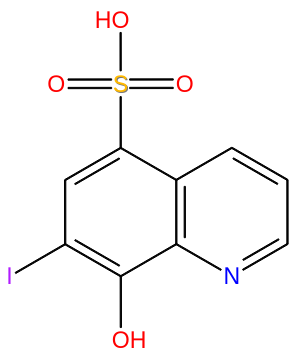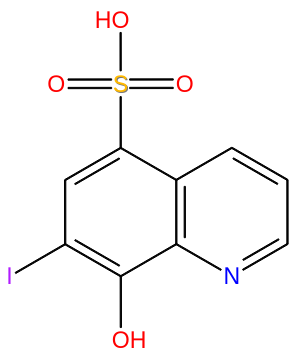11043 | PS(AS) | Inactive | Low | Inactive | Low |
| BENZETHONIUM CHLORIDE | 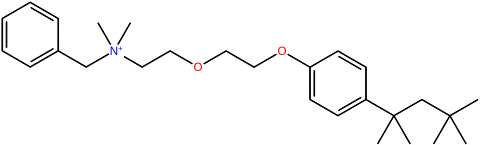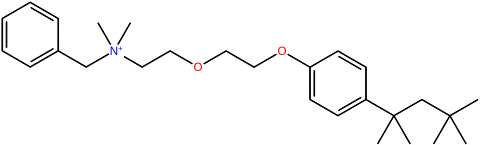2335, 8478 | PS(AD) | 1.53 | Moderate | 9.52 | Moderate |
| SALINOMYCIN, SODIUM | 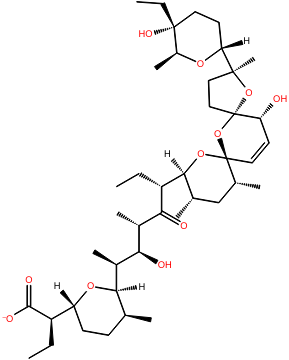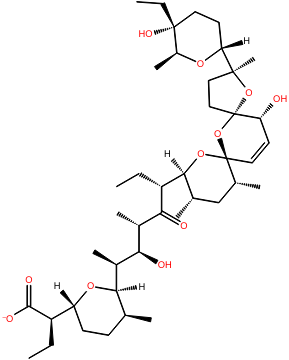3085092, 45479870 | PS(AD) | NT |  | NT |  |
| PROTRYPTYLINE HYDROCHLORIDE | 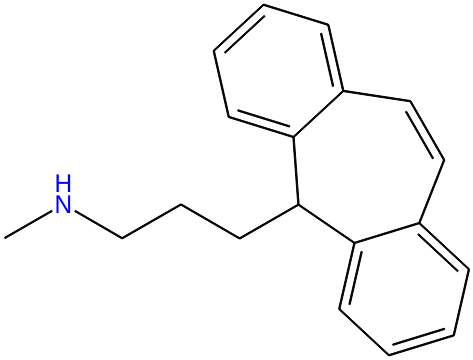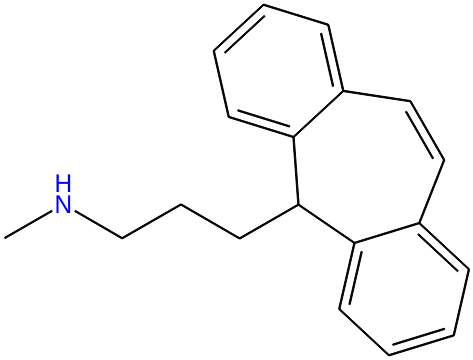4976 | PS(AS) | NT |  | NT |  |
| PERHEXILINE MALEATE | 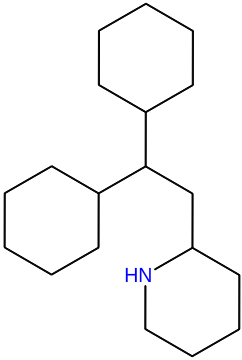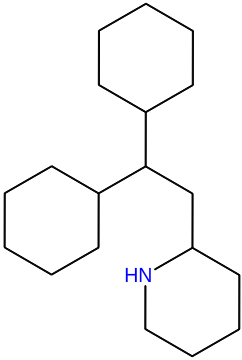4746, 5284439 | PS(AD) | 2.66 | Moderate | 16.93 | Low |
| PYRANTEL PAMOATE | 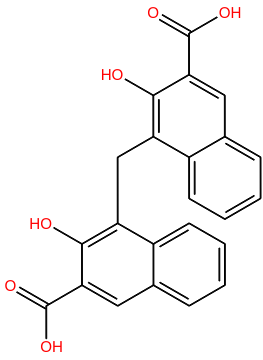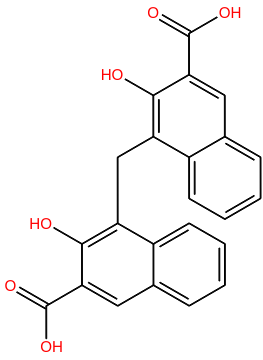708857, 5281033 | PS(AD) | NT |  | NT |  |
| NICOTINE DITARTRATE | 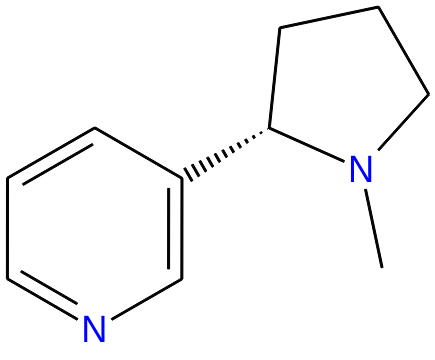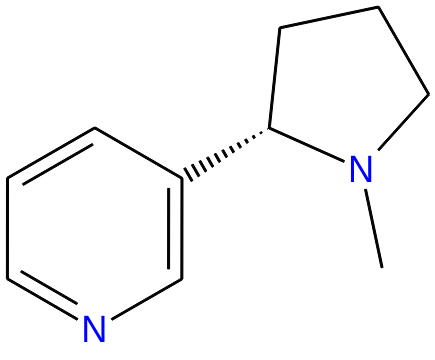89594, 5702296 | PS(AS) | Inactive | Low | Inactive | Low |
| MOXIFLOXACIN HYDROCHLORIDE | 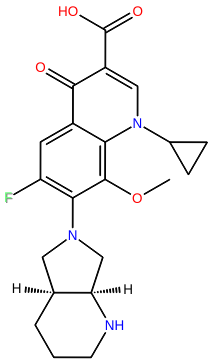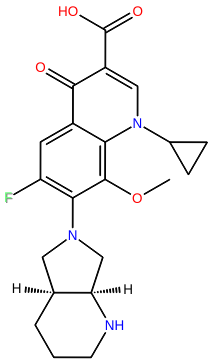101526, 152946 | PS(AS) | Inactive | Low | Inactive | Low |
| DICHLORVOS | 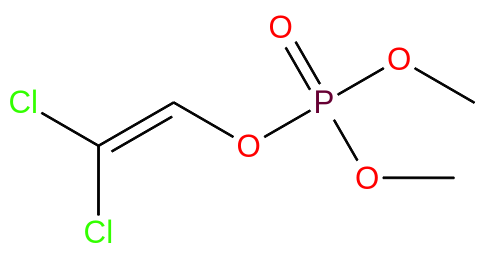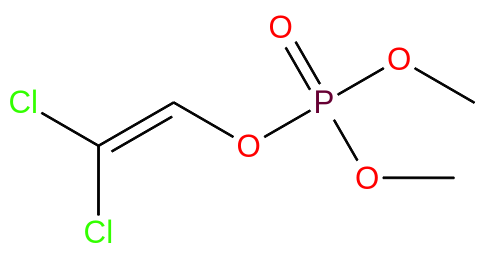3039 | PS(AD) | 9.44 | Moderate | Inactive | Low |
| NIMODIPINE | 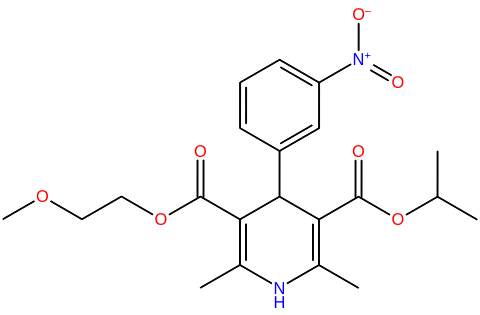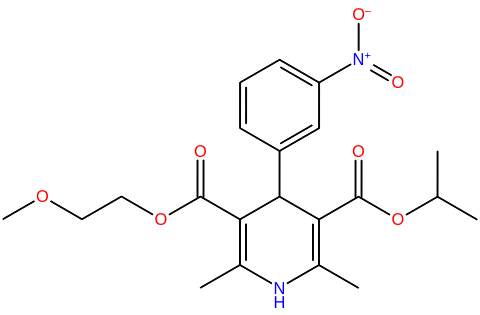4497 | PS(AS) | Inactive | Low | 10.68 | Low |
| BISACODYL | 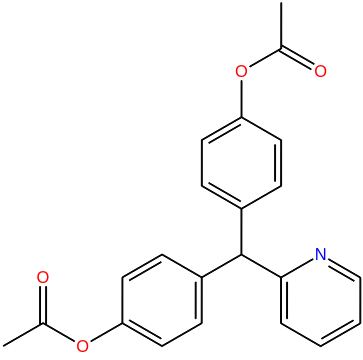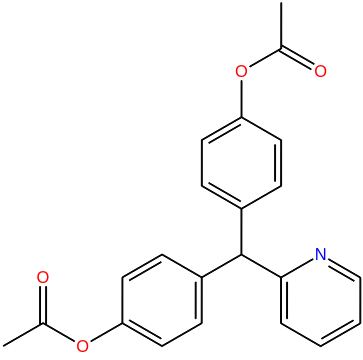2391 | PS(AS) | Inactive | Low | Inactive | Low |
| THIABENDAZOLE | 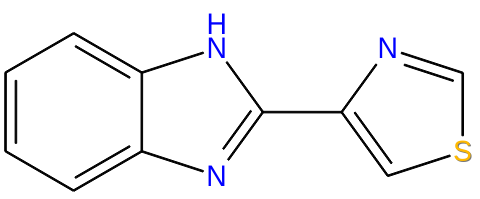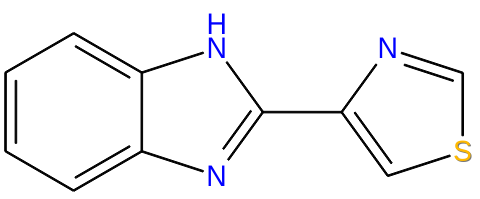5430 | PS(AD) | Inactive | Low | 27.53 | Low |
| MORANTEL CITRATE | 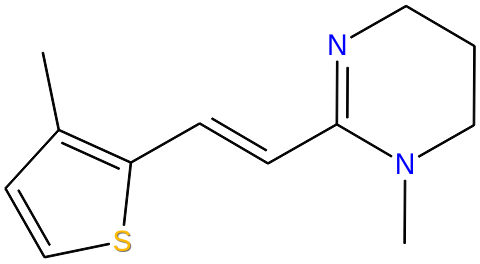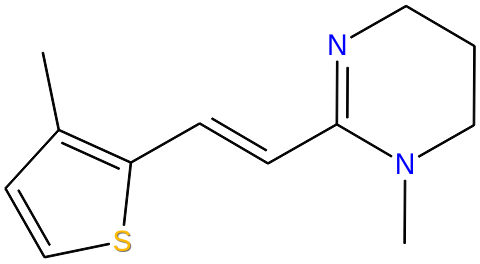5353792, 5702276 | PS(AS) | NT |  | NT |  |
| HEXETIDINE | 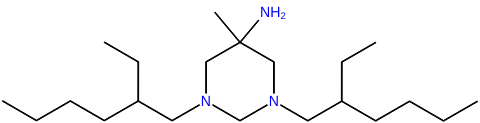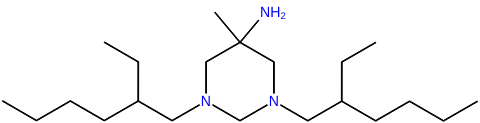3607 | PS(AD) | 10.12 | Low | 8.49 | Moderate |
| QUINAPRIL HYDROCHLORIDE | 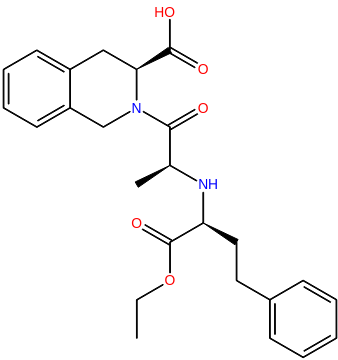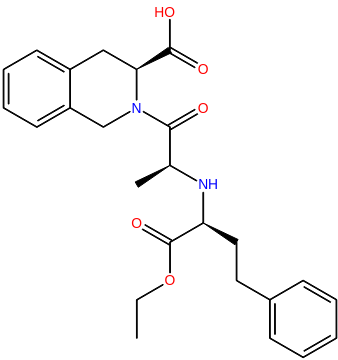54891, 54892 | PS(AS) | Inactive | Low | Inactive | Low |
| RONIDAZOLE | 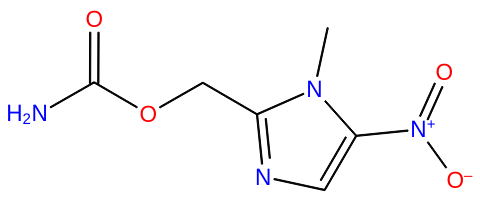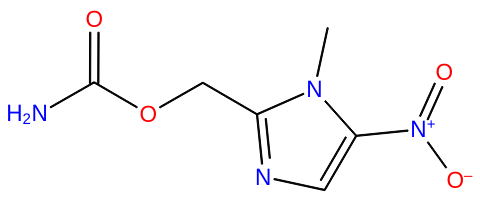5094 | PS(AS) | Inactive | Low | Inactive | Low |
| PHYSOSTIGMINE SALICYLATE | 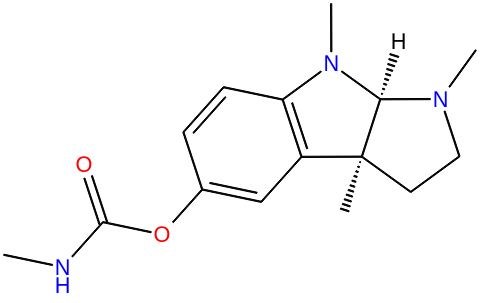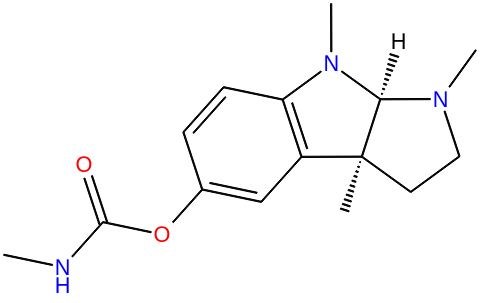657348, 16212981 | PS(AS) | NT |  | NT |  |

^a^ Parent CIDs for salt-free forms are provided where appropriate

^b^ NG: Nematode-general, PS(AD): Parasite-selective (adult and E2L), PS(AS): Parasite-selective (adult-selective)

^c^ Data are from https://pubchem.ncbi.nlm.nih.gov/bioassay/743012

^d^ High: IC_50_ ≤ 1 uM, Moderate: 1 uM < IC_50_ < 10 uM, Low: IC_50_ ≥ 10 uM

^e^ Data are from https://pubchem.ncbi.nlm.nih.gov/bioassay/1224886

^f^ NT: not tested

Table-S3(Aroian). Acute toxicity and cytotoxicity data for broad-spectrum (adult hookworm and whipworm) actives.

| 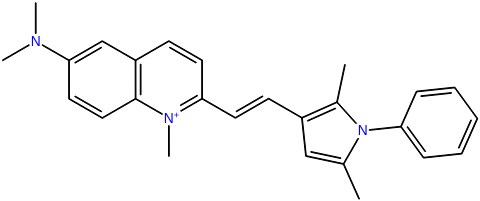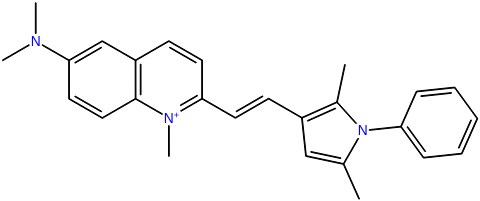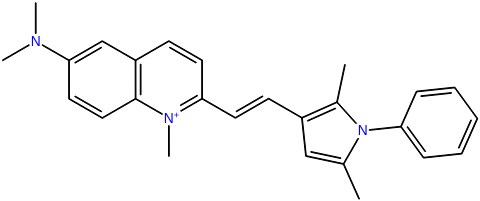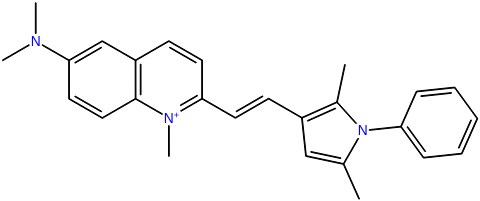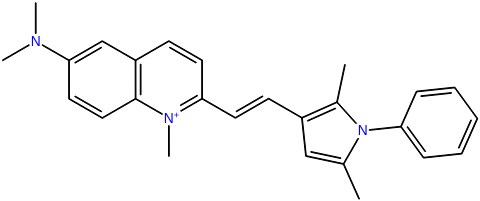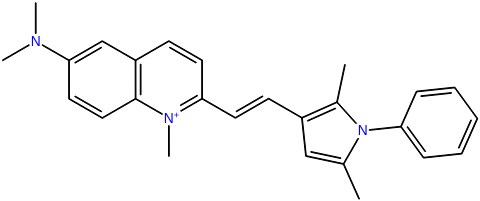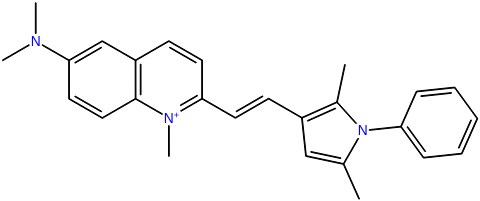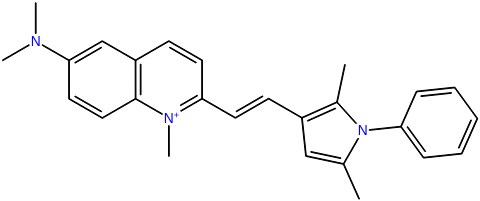 | ***A. ceylanicum* Adult 30uM** | ***A. ceylanicum* E2L 30 µM** | ***C. elegans* L4 30 µM** | ***T. muris*, 100 µM** | **Oral LD50^a^ (mouse), mg/kg** | **PubChem CID** | **Selectivity Class** | **DT40 IC50, uM (PubChem AID743012)** | **DT40 Cytotox. Ranking** | **HEK293 IC50, uM (PuChem AID1224886)** | **HEK293 Cytotox. Ranking** |
| --- | --- | --- | --- | --- | --- | --- | --- | --- | --- | --- | --- |
| **PHENYLMERCURIC ACETATE** | * | * | * | * | 13 | 16682730 | NG | 0.11 | High | 0.24 | High |
| **IVERMECTIN** | * | * | * | * | 25 | 3085416, 11957587 | NG | NT |  | NT |  |
| **MONENSIN SODIUM** | * |  |  | * | 44 | 441145. 23667299 | PS(AS) | 0.47 | High | NT |  |
| **DICHLORVOS** | * | * |  | * | 61 | 3039 | PS(AD) | 9.44 | Moderate | Inactive | Low |
| **NICOTINE DITARTRATE** | * |  |  | * | 65 | 89594, 5702296 | PS(AS) | Inactive | Low | Inactive | Low |
| **HEXACHLOROPHENE** | * | * |  | * | 67 | 3598 | PS(AS) | 3.05 | Moderate | 3.88 | Moderate |
| **PENFLURIDOL** | * | * | * | * | 87 | 33630 | NG | 4.22 | Moderate | 9.52 | Moderate |
| **THIMEROSAL** | * | * | * | * | 91 | 16684434 | NG | 0.17 | High | 1.10 | Moderate |
| **GENTIAN VIOLET** | * | * | * | * | 96 | 11057 | NG | 0.19 | High | 5.47 | Moderate |
| **CETYLPYRIDINIUM CHLORIDE** | * | * | * | * | 108 | 2683, 31239 | NG | 0.43 | High | 17.37 | Low |
| **LEVAMISOLE HYDROCHLORIDE** | * | * | * | * | 223 | 26879, 27944 | NG | Inactive | Low | Inactive | Low |
| **PROTRYPTYLINE HYDROCHLORIDE** | * |  |  | * | 269 | 4976 | PS(AS) | NT |  | NT |  |
| **ECONAZOLE NITRATE** | * | * |  | * | 462 | 3198, 68589 | PS(AD) | 7.50 | Moderate | 21.79 | Low |
| **BENZALKONIUM CHLORIDE** | * | * |  | * | 919 | 23705 | PS(AD) | 0.42 | High | 3.01 | Moderate |
| **HEXETIDINE** | * | * |  | * | 1520 | 3607 | PS(AD) | 10.12 | Low | 8.49 | Moderate |
| **SULCONAZOLE NITRATE** | * | * | * | * | 2475 | 5318, 65495 | NG | 10.12 | Low | 16.93 | Low |
| **PERHEXILINE MALEATE** | * | * |  | * | 2641 | 4746, 5284439 | PS(AD) | 2.66 | Moderate | 16.93 | Low |
| **PYRANTEL PAMOATE** | * | * | * | * | 4090 | 708857, 5281033 | PS(AD) | NT |  | NT |  |
| **PARAROSANILINE PAMOATE** | * | * |  | * | 5000 | 11292, 11293 | PS(AD) | 9.69 | Moderate | 3.47 | Moderate |

^a^ ChemID*plus* database of TOXNET (https://chem.nlm.nih.gov/chemidplus/)


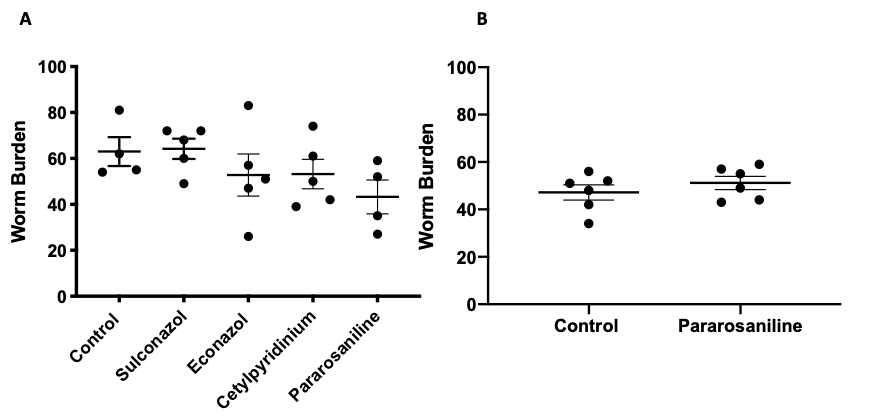


Figure-S1(Aroian) ***in vivo* activity against *A. ceylanicum* infections in hamsters.**
